# Probiotics for the Treatment of Docetaxel-Related Weight Gain of Breast Cancer Patients—A Single-Center, Randomized, Double-Blind, and Placebo-Controlled Trial

**DOI:** 10.3389/fnut.2021.762929

**Published:** 2021-12-02

**Authors:** Zhang Juan, Zhang Qing, Liang Yongping, Liyuan Qian, Wei Wu, Yanguang Wen, Jianbin Tong, Boni Ding

**Affiliations:** ^1^Department of Breast Surgery, Tangshan People's Hospital, Tangshan, Hebei, China; ^2^Department of Breast and Thyroid Surgery, Third Xiangya Hospital, Central South University, Changsha, China; ^3^Department of Medical Imaging (Ultrasound), Tangshan Central Hospital, Tangshan, China; ^4^Hunan Province Key Laboratory of Brain Homeostasis, Third Xiangya Hospital, Central South University, Changsha, China; ^5^Center for Experimental Medicine, Third Xiangya Hospital, Central South University, Changsha, China; ^6^Department of Anesthesiology, Third Xiangya Hospital, Central South University, Changsha, China

**Keywords:** docetaxel-related weight gain, breast cancer, docetaxel-based chemotherapy, gut microbiota, metabolism

## Abstract

**Background:** Docetaxel is an important chemotherapy-agent for breast cancer treatment. One of its side-effects is weight gain, which increases the all-cause mortality rate. Considering gut microbiota is one important factor for weight regulation, we hypothesized that probiotics could be potentially used to reduce the docetaxel-related weight gain in breast cancer patients.

**Methods:** From 10/8/2018 to 10/17/2019, 100 breast cancer (Stage I-III) patients underwent four cycles of docetaxel-based chemotherapy were enrolled and randomly assigned to receive probiotics (*Bifidobacterium longum, Lactobacillus acidophilus*, and *Enterococcus faecalis*) or placebo (supplementary material of the probiotics capsule) treatment for 84 days with three capsules per time, twice/day. The primary outcome: the changes in body weight and body-fat percentage of the patients were measured by a designated physician using a fat analyzer, and the secondary outcomes: the fasting insulin, plasma glucose, and lipids were directly obtained from the Hospital Information System (HIS); The metabolites were measured using liquid chromatography coupled with tandem mass spectrometry (LC-MS/MS); The fecal microbiome was analyzed using bacterial 16S ribosomal RNA (rRNA) gene sequence. All indicators were measured 1 day before the first cycle of docetaxel-based chemotherapy and 21 days after the last cycle of docetaxel-based chemotherapy.

**Results:** Compared with the placebo group, the probiotic group showed significantly smaller changes in body weight (Mean [SD] 0.77 [2.58] vs. 2.70 [3.08], *P* = 0.03), body-fat percentage (Mean [SD] 0.04 [1.14] vs. 3.86 [11.09], *P* = 0.02), and low density lipoprotein (LDL) (Mean [SD]−0.05[0.68] vs. 0.39 [0.58], *P* = 0.002). Moreover, five of the 340 detected plasma metabolites showed significant differences between the two groups. The change of biliverdin dihydrochloride (*B* = −0.724, *P* = 0.02) was inverse correlated with weight gain. One strain of the phylum and three strains of the genus were detected to be significantly different between the two groups. Also, the changes of *Bacteroides* (*B* = −0.917, *P* < 0.001) and *Anaerostipes* (*B* = −0.894, *P* < 0.001) were inverse correlated with the change of LDL.

**Conclusions:** Probiotics supplement during docetaxel-based chemotherapy for breast cancer treatment may help to reduce the increase in body weight, body-fat percentage, plasma LDL, and minimize the metabolic changes and gut dysbacteriosis.

**Clinical Trial Registration:** http://www.chictr.org.cn/showproj.aspx?proj=24294, ChiCTR-INQ-17014181.

## Introduction

The population of obesity in the world is growing rapidly (1). The impact of obesity on health has been revealed more clearly. In addition to metabolic diseases, obesity is closely related to the occurrence of a variety of malignant tumors (2), as well as a worse prognosis (3).

Breast cancer is the most common malignancy in women (4), and chemotherapy is one of its main treatments. One of the most important chemotherapy-agent for breast cancer is docetaxel which may induce weight gain and the increase of blood glucose, triglyceride, and insulin (5). Almost 50–96% of the breast cancer patients who underwent chemotherapy showed varying degrees of weight gain (6). It is also known that breast cancer patients who gained weight (especially >10% of their body weight) during the treatment showed increased recurrence risk as well as all-cause mortality rates (7). Furthermore, obesity-related diseases (e.g., hypertension, diabetes, cardiovascular and cerebrovascular diseases, gallbladder diseases, etc.) significantly decrease the quality of life of breast cancer patients (8, 9).

The mechanism of docetaxel-related weight gain is unclear yet. It may be related to the changes of food intake, basal metabolic rate, physical activity, menstrual status, and hormone levels (10–13). Besides, it is suggested that the host gut microbiota is associated with obesity and metabolic syndrome (14). Therefore, probiotics supplement has the potential therapeutic effects on weight gain, metabolic syndrome, and chronic inflammation state (15). Previous research showed that the most investigated probiotics with antiobesity potential are those of *Lactobacillus spp*., *Bifidobacterium spp*., and *Enterococcus spp*. (16, 17). Also, the feeding of soy milk fermented by probiotic starter which consists of *Streptococcus thermophilus, Lactobacillus acidophilus* LA-5, and *Bifidobacterium bifidum* Bb-12 which could helped to decrease the rate of cholesterol and triglyceride (18). Thus, we hypothesized that supplement of this probiotic multi-strain formulation during docetaxel-based chemotherapy could potentially reduce the docetaxel-related weight gain in breast cancer patients.

## Materials and Methods

### Participants

**Figure 1:**
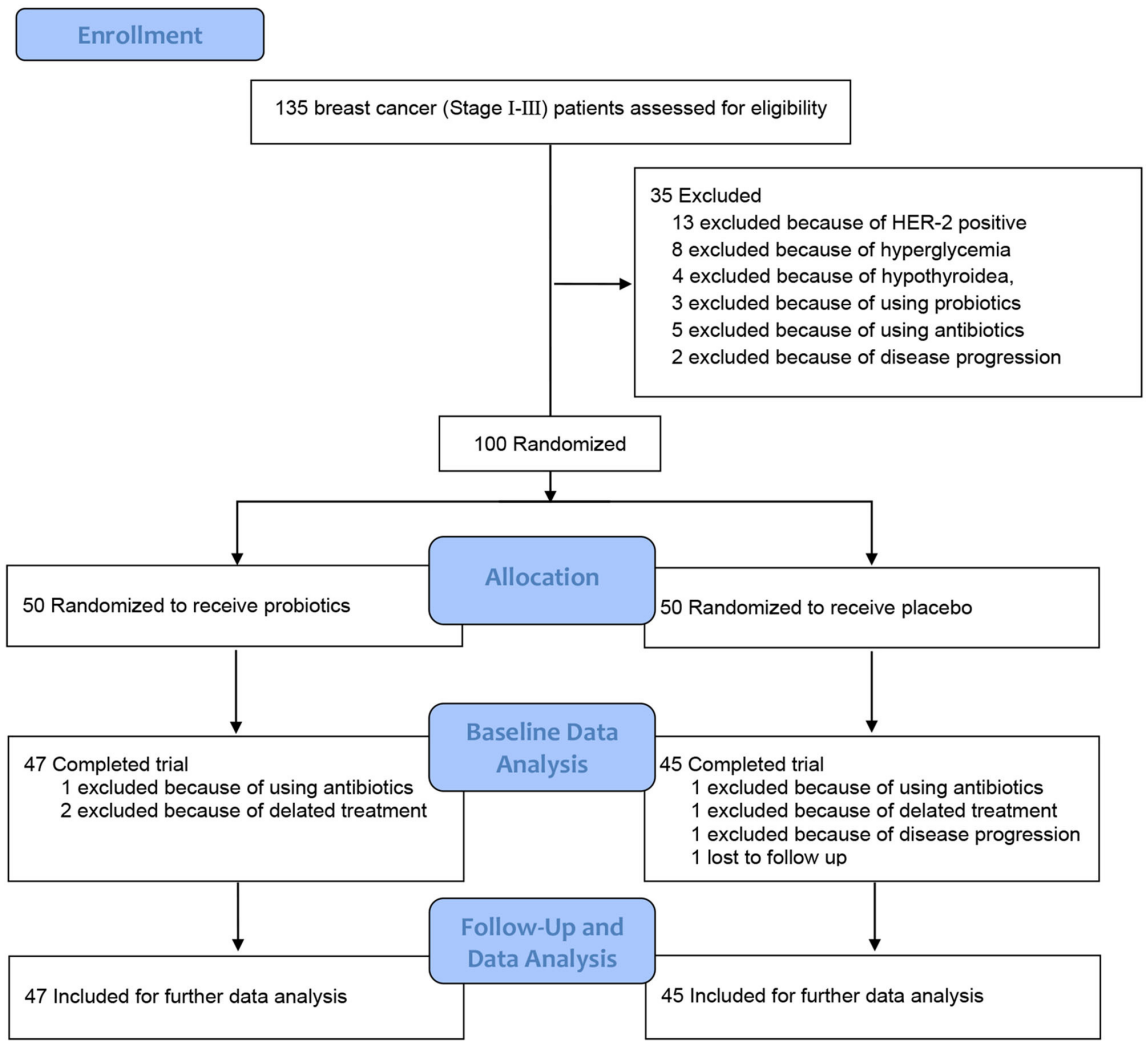
Study flow chart.

To verify whether probiotics supplement during docetaxel-based chemotherapy can reduce docetaxel-related weight gain, a randomized, double-blind, placebo-controlled clinical trial was carried out. The prospective trial protocol (Supplementary Material 1) was approved by the Ethics Committee (Ethical approval number: 2018-S285), Third Xiangya Hospital, Central-South university, and registered on 12/27/2017 in Chinese Clinical Trial Registry (ChiCTR-INQ-17014181) [http://www.chictr.org.cn/index.aspx]. From 10/8/2018 to 10/17/2019, patients diagnosed with invasive breast cancer stages I to III (AJCC 8th edition) who were admitted to the Department of Breast and Thyroid Surgery, Third Xiangya Hospital and needed docetaxel-based chemotherapy, were screened for potential recruitments after obtaining written informed consent (Figure 1). The inclusion criteria were newly diagnosed breast cancer (Stage I-III) patients who had operation and required docetaxel-based chemotherapy (four cycles of epirubicin at 100 mg/m^2^ and cyclophosphamidum at 600 mg/m^2^ followed by four cycles of docetaxel at 100 mg/m^2^ [EC-T]), between 20 and 60 years old of age, without immune system diseases, and agreed to participate. Patients were excluded when met any of the following criteria: human epidermal growth factor receptor-2 (HER-2) was positive; advanced breast cancer patients; with other malignant tumors; with diabetes; thyroid dysfunction or after thyroidectomy; pituitary tumor; adrenal gland tumor and other diseases that seriously affected metabolism; history of ovariectomy, thyroidectomy, pituitary surgery, adrenal surgery and other operations that seriously affected endocrine function; history of using antidepressants, weight-loss medications or other medications that promoted weight gain or metabolic loss; participated in or plan to participate in diet or exercise weight loss programs; used antibiotics, probiotics, or gastrointestinal motility drugs within 3 months before admission or during the study; alcoholic or drug addicts; participating in other clinical trials; refused to participate; could not cooperate with the treatment.

### Primary and Secondary Outcomes

#### Primary Outcome

The primary outcome was the changes in body weight and body-fat percentage of the patients enrolled which were measured 1 day before the first cycle of docetaxel-based chemotherapy and 21 days after the last cycle of docetaxel-based chemotherapy (in the following contents, we would use the term “before docetaxel-based chemotherapy” and “after docetaxel-based chemotherapy,” respectively). The body weight of the patients (with only underwear, empty stomach and bladder) was measured by a designated physician (0.1 kg precision). The body-fat percentage of patients was detected (0.1% precision) using a fat analyzer (Mi Body Composition Scale XMTZC02HM, Xiaomi Technology Co. LTD, China). The data were collected through duplicate measurements and the average value was calculated.

#### Secondary Outcomes

1) The fasting blood samples of patients enrolled were collected before and after docetaxel-based chemotherapy. The collected samples were maintained at 4°C and were transported to the clinical laboratory of the hospital immediately for measurement. The data of fasting plasma insulin, plasma glucose and lipids were directly obtained from the Hospital Information System (HIS).
2) Plasma metabolites: the fasting blood samples were collected as mentioned above except using anticoagulant (EDTA-K2) tubes. Plasma and lymphocytes isolated from the blood samples were frozen in the refrigerator (−80°C). Thirty patients (15 patients per group) were randomly selected before and after docetaxel-based chemotherapy, their fasting blood samples (a total of 60 samples, 30 samples per group) were measured using liquid chromatography coupled with tandem mass spectrometry (LC-MS/MS) offered by Biotree biomedical technology co. Ltd, Shanghai, China.
3) Fecal microbiome compositions: the stool samples were collected using disposable sterile stool specimen box before and after docetaxel-based chemotherapy. The collected samples were transported to the laboratory at low temperature within 4 h after collected and aliquoted into 2–3 cryopreservation tubes, and stored in the refrigerator (−80°C). Thirty patients (15 patients per group) were randomly selected and their stool samples (a total of 60 samples, 30 samples per group) were analyzed using bacterial 16S ribosomal RNA (rRNA) gene sequence (Majorbio pharmaceutical technology co. LTD, Shanghai, China).

### Other Clinical Data Collection

Clinical characteristics including demographics such as age, height, molecular subtype, and disease stage were obtained from the medical record. Toxic and side effects including hematological, gastrointestinal, alopecia, heart, kidney function indicators were evaluated every cycle (divided into 0–4) according to World Health Organization (WHO) classification standards of antitumor drug adverse reaction.

### Sample Size Calculations

In our preliminary study of 40 patients (20 in the probiotics group and 20 in the placebo group), the proportion of patients who gained weight is 85% in the placebo group, and 40% in the probiotics group. With a significance set at 0.05 and power at 90%, a significant difference is detected according to the equation: $n=\frac{2{\left(\mu_{\alpha }+\mu_{\beta }\right)}^{2}p\left(1-p\right)}{\delta^{2}}$, where δ = (P_1_-P_2_), *p* = (P_1_+P_2_)/2, and P_1_ and P_2_ are the positive rates of the placebo group and the probiotics group, respectively. Although 88 patients were needed, considering a loss to follow-up rate of around 10%, a total of 100 patients were enrolled.

### Randomization and Masking

A researcher who was not involved in data management or statistical analysis performed randomization with a 1:1 ratio using SPSS 19.0 software. The randomized numbers written in a paper were sealed in an envelope and stored until the study ended. Patients enrolled received probiotics or placebo from a nurse who was not involved in data management or statistical analyses according to the assignment. During the study, the patients, the physicians who were involved in patients' and the researcher who conducted the follow-up, were all blinded. If any unexpected things happened to the enrolled patients, physicians could unmask the treatment assignment, or remove the patient from the study.

### Drugs

The dose of docetaxel (Jiangsu Hengrui Medicine CO., LTD., Jiangsu, China) was 100 mg/m^2^, which was administered as a cycle of 21 days for a total of four treatment cycles. Before docetaxel administration, a total of 10 mg dexamethasone were administered intravenously. The probiotic capsules (BIFICO, Sine Pharmaceuticals, Shanghai, China, batch number: 04720190507) contain *Bifidobacterium longum* (≥1.0 × 10^7^ CFU/210 mg), *Lactobacillus acidophilus* (≥1.0 × c10^7^ CFU/210 mg), *Enterococcus faecalis* (≥1.0 × 10^7^ CFU/210 mg), and supplementary material (degreased milk powder, maltodextrin, sugarcane fruit oligosaccharide, food essence, ethanol, and pure water). The placebo capsules (Sine Pharmaceuticals, Shanghai, China, batch number: SZ04720190501) contain only the supplementary material (degreased milk powder, maltodextrin, sugarcane fruit oligosaccharide, food essence, ethanol, and pure water) and have similar shape, size, and smell to the probiotic capsules. The probiotics and placebo capsules were kept in refrigerator at 2–8°C to maintain the stability and quality. Both probiotic and placebo groups received 3 capsules (0.84 g) twice/day (30 min after lunch and dinner) throughout the treatment period.

### Statistical Analysis

For both per-protocol analysis (PP) and intent-to-treat analysis (ITT), the body weight, BMI, and body-fat percentage before and after docetaxel-based chemotherapy were compared within the group and between the two groups using an unpaired *t*-test, while the changes of the body weight, BMI, and body-fat percentage were compared between the two groups using an unpaired *t*-test. Last observation carry forward method was used for the ITT analysis. Covariance analysis was used to adjust the imbalances. All other variables were compared between the two groups using an unpaired *t*-test except when there was not a normal distribution (such as the data for molecular subtype and disease stage), Mann-Whitney test was used instead. All other data collected at multi-time points were analyzed with analysis of variance (ANOVA) followed by Scheffe's Test. For the changes of plasma metabolites, Chi square test was used. The Binary Logistic regression, and Pearson correlation coefficient were used to analyze the association of microbiota and metabolites with the primary and secondary outcome. For the gut microbiota, sample alpha-diversity was summarized using Chao1 estimator and Shannon index, and beta-diversity was analyzed using Principal Component Analysis (PCA). To compare the gut microbial community, Wilcoxon rank-sum test was used. A 2-tailed *P* < 0.05 was considered to be a statistical significance.

## Results

### Demographics of Patients Studied

From 10/8/2018 to 10/17/2019, 135 breast cancer patients (Stage I-III) were screened for potential recruitments as mentioned above. Of those, 13 patients (9.6%) were excluded because of HER-2 positive, eight patients (5.9%) were excluded because of hyperglycemia, four patients (3.0%) were excluded because of hypothyroidea, three patients (2.2%) were excluded because of taking probiotics within 3 months before admission, five patients (3.7%) were excluded because of taking antibiotics within 3 months before admission, and two patients (1.5%) were excluded because of disease progression. A total of 100 patients were enrolled and randomly assigned to receive either probiotics (*n* = 50 [50%]) or placebo (*n* = 50 [50%]) (Figure 1). The demographics and clinical characteristics of patients in the two groups are showed in Table 1. Ultimately, 92 patients (92%) completed the study with 47 (94%) from the probiotics group and 45 (90%) from the placebo group. The withdrawal rates were similar in the two groups and the overall withdrawal rate was 8% (8 of 100) among which 2 (2%) were suspended due to using antibiotics, 3 [3%] due to delayed treatment, 1 [1%] due to disease progression, 1 [1%] due to cerebrovascular accident death, and 1 [1%] due to treatment abandonment. All baseline and follow-up data from 92 patients (92%) were included for further per-protocol analysis.

**Table 1:** The baseline demographic and clinical characteristics of patients.

**Variable**	**Mean (SD)**	
	**Probiotics group^a^ (*n* = 47)**	**Placebo group^a^ (*n* = 45)**
Age, mean (SD), y	45.48 (8.37)	46.83 (8.25)
Height, mean (SD), cm	157.08 (4.73)	158.29 (4.87)
Weight, mean (SD), kg	56.11 (6.53)	58.05 (7.29)
BMI, mean (SD)	22.66 (2.34)	23.11 (2.81)
BFP, mean (SD), %	31.95 (4.76)	32.33 (5.42)
Molecular subtype, No. (%)		
Luminal A	13 (28)	10 (22)
Luminal B	18 (38)	19 (42)
HER-2-Postive	10 (21)	8 (18)
Tri-Negative	6 (13)	8 (18)
Disease stage (AJCC 8th edition, TNM), No. (%)		
I	10 (21)	8 (18)
II	29 (62)	24 (53)
III	8 (17)	13 (29)

*BMI, Body Mass Index; BFP, Body-fat Percentage; Luminal A, Estrogen receptor positive [ER (+)], and Progesterone receptor ≥ 20%, Human epidermal growth factor receptor-2 negative [HER-2 (-)], and Ki-67≤ 20%; Luminal B, Estrogen receptor positive [ER (+)], and/or Progesterone receptor positive [PR (+)], Human epidermal growth factor receptor-2 negative [HER-2 (-)], and Ki-67>20%, or Estrogen receptor positive [ER (+)], and/or Progesterone receptor positive [PR (+)], Human epidermal growth factor receptor-2 positive [HER-2 (+)]; HER-2-Postive, Estrogen receptor negative [ER (-)], and Progesterone receptor negative [PR (-)], Human epidermal growth factor receptor-2 positive [HER-2 (+)]; Tri-Negative, Estrogen receptor negative [ER (-)], and Progesterone receptor negative [PR (-)], Human epidermal growth factor receptor-2 negative [HER-2 (-)]; AJCC, American Joint Committee on Cancer; TNM, Tumor size (T), Nodal status (N), and Metastasis (M)*.

a *No differences between the two groups on any variable based on t-test, or Mann-Whitney test, P > 0.05*.

### Probiotics Alleviated the Change of Body Weight, BMI, and Body-Fat Percentage

On the baseline and after docetaxel-based chemotherapy, the body weight, BMI, and body-fat percentage of the patients showed no significant difference between the two groups. However, in the placebo group, the changes of body weight (*P* = 0.03), BMI (*P* = 0.04), and body-fat percentage (*P* = 0.02) were all significantly higher than those in the probiotics group (Table 2). Throughout the treatment period, the body weight (*P* = 0.001), BMI (*P* = 0.001), and body-fat percentage (*P* = 0.001) of patients from the placebo group increased significantly, while the body weight (*P* = 0.15), BMI (*P* = 0.13), and body-fat percentage (*P* = 0.86) of patients from the probiotics group stayed stable (Table 3). By using covariance analysis, none of the patients' indexes before the first cycle of docetaxel administration had significant effect on the results (Supplementary Table 2). And the indicators of the withdrawal patients had no effects on the results also (Supplementary Tables 3–5).

**Table 2:** The status of body weight, BMI, and BFP of participants enrolled over time.

**Results**	**Probiotics *n* = 47 Mean (SD)**	**Placebo *n* = 45 Mean (SD)**	***P*-value**
Weight, mean (SD), kg	56.50 (6.29)	59.40 (7.20)	0.15
BMI, mean (SD)	22.83 (2.17)	23.67 (2.73)	0.25
BFP, mean (SD), %	31.99 (4.67)	36.19 (10.65)	0.08
Weight Change, kg	0.77 (2.58)	2.70 (3.08)	0.03
BMI Change	0.17 (0.54)	0.56 (0.62)	0.04
BFP Change, %	0.04 (1.14)	3.86 (11.09)	0.02

*BMI, Body Mass Index; BFP, Body-fat Percentage*.

*The differences between the two groups based on t-test*.

**Table 3:** The changes of body weight, BMI, and BFP of participants enrolled over time.

**Results**	**Probiotics** ***n*** **=** **47 Mean (SD)**			**Placebo** ***n*** **=** **45 Mean (SD)**		
	**Pre**	**Post**	***P*^a^-value**	**Pre**	**Post**	***P*^b^-value**
Weight, kg	56.11 (6.53)	56.20 (6.29)	0.15	58.05 (7.29)	59.40 (7.19)	0.001
BMI	22.66 (2.34)	22.83 (2.17)	0.13	23.11 (2.81)	23.67 (2.73)	0.001
BFP, %	31.94 (4.76)	31.99 (4.67)	0.86	32.33 (5.42)	36.19 (10.65)	0.001

*BMI, Body Mass Index; BFP, Body-fat Percentage; Pre, 1 day before the first cycle of docetaxel administration; Post, 21 days after the last cycle of docetaxel administration*.

a *The differences in the probiotics group 1 day before the first cycle of docetaxel administration and 21 days after the last cycle of docetaxel administration based on t-test*.

b *The differences in the placebo group 1 day before the first cycle of docetaxel administration and 21 days after the last cycle of docetaxel administration based on t-test*.

### Probiotics Maintained the Levels of LDL, TC, and GLU

The levels of plasma low density lipoprotein (LDL) in the placebo group was significantly higher than that in the probiotics group after docetaxel-based chemotherapy (*P* = 0.005), and the changes of plasma total cholesterol (TC) (*P* = 0.048), glucose (GLU) (*P* = 0.04), and LDL (*P* =0.002) throughout the treatment period showed significant difference between the two groups. There were no significant differences in other measurements between the two groups before or after docetaxel administration (Supplementary Table 1).

### Probiotics Modulated Docetaxel-Induced Metabolic Changes

**Figure 2:**
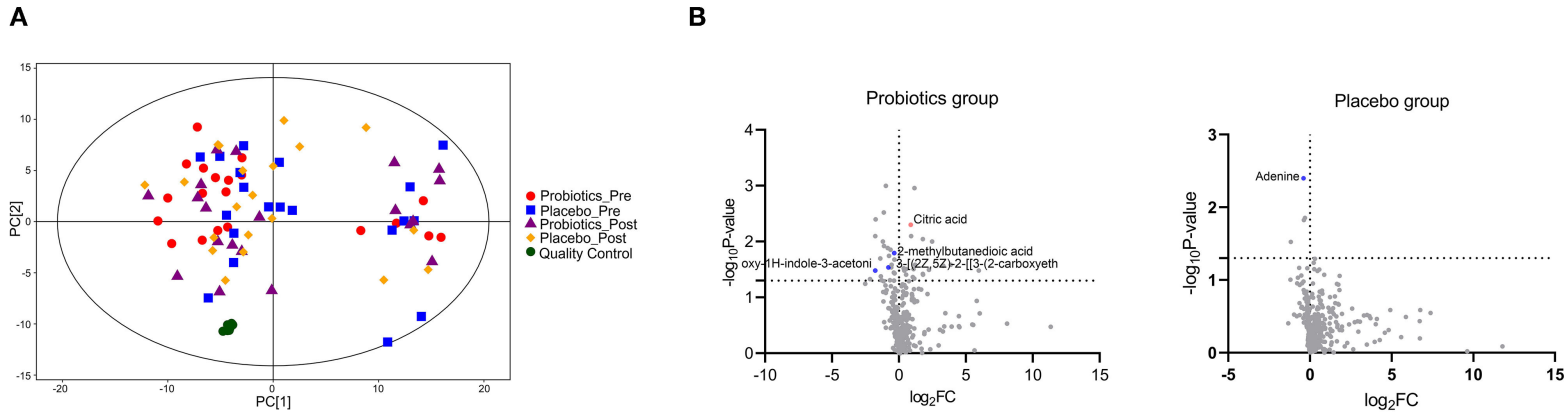
Comparison of the plasma metabolites in the two groups (a total of 60 sample from 15 patients/group 1 day before the first cycle of docetaxel administration and 21 days after the last cycle of docetaxel administration.). **(A)** The principal component analysis (PCA) scores plots constructed with metabolites of samples of patients. **(B)** The differences between the two groups on metabolites based on Mann-Whitney test. Metabolites with significant intergroup difference throughout the treatment period (up-regulation: red points; down-regulation: blue points; no significant intergroup difference: gray points).

Among the 340 detected metabolites, the changes of five metabolites throughout the treatment period showed a significant intergroup difference (Figure 2; Supplementary Table 6). The metabolic pathways involved amino acids metabolism (e.g., Tyrosine metabolism), lipid metabolism (primary bile acid biosynthesis), carbohydrate metabolism (citrate cycle), and nucleotide metabolism (purine metabolism). The changes of Biliverdin dihydrochloride (*B* = −0.724, *P* = 0.02) were inverse correlated with the weight gain.

### Probiotics Improved Gut Microbial Composition

**Figure 3:**
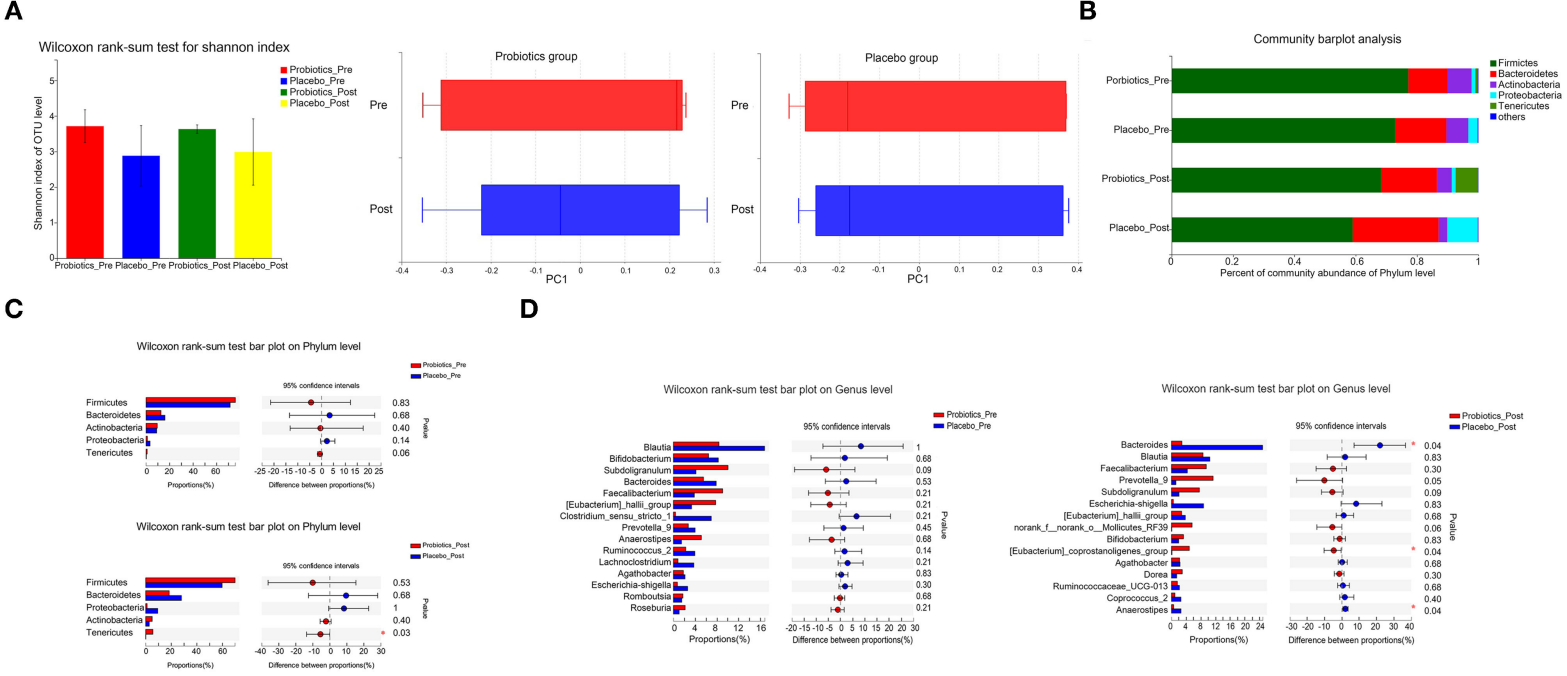
Comparison of the gut microbiota in the probiotics group and the placebo group (a total of 60 samples from 15 patients/group 1 day before the first cycle of docetaxel administration and 21 days after the last cycle of docetaxel administration). **(A)** α-diversity (Shannon) and β-diversity (PCA) of gut microbiota. **(B)** The proportion of gut microbiota composition of the patients in the two groups on the Phylum level 1 day before the first cycle of docetaxel administration and 21 days after the last cycle of docetaxel administration. **(C)** The gut microbial community of the patients in the two groups on the Phylum level 1 day before the first cycle of docetaxel administration and 21 days after the last cycle of docetaxel administration. *P < 0.05. **(D)** The gut microbial community of the patients in the two groups on the Genus level 1 day before the first cycle of docetaxel administration and 21 days after the last cycle of docetaxel administration. *P < 0.05. The differences between the two groups on the proportion of gut microbiota composition on both the Phylum level and the Genus Level based on Wilcoxon rank-sum test.

Before and after docetaxel-based chemotherapy, gut microbiota of the two groups showed no statistical difference in Alpha diversity or Beta diversity (Figure 3A). On both Phylum and Genus levels, the gut microbial community of patients in the two groups was similar before docetaxel-based chemotherapy (Figures 3B–D). However, after docetaxel-based chemotherapy, the relative abundance of *Tenericutes* (Mean [SD] 5.62 [9.73] vs. 0.07 [0.10], *P* = 0.03) on phylum level in the probiotics group was signficantly higher than that in the placebo group. The relative abundance of *Bacteroide*s (Mean [SD] 2.81 [1.53] vs. 24.77 [19.13], *P* = 0.04), [Eubacterium]_*coprostanoligenes_group* (Mean [SD] 4.84 [6.26] vs. 0.12 [0.16], *P* = 0.04), and *Anaerostipes* (Mean [SD] 0.57 [0.30]vs. 2.58 [1.91], *P* = 0.04) on Genus level also showed significant differences in the two groups after docetaxel-based chemotherapy (Figures 3C,D). The changes of *Bacteroides* (*B* = −0.917, *P* < 0.001) and *Anaerostipes* (*B* = −0.894, *P* < 0.001) were inverse correlated with the change of LDL.

In addition, compared with the placebo group, the incidence of constipation in the probiotics group was significantly lower, while no severe emesis or constipation (≥Grade 3) were observed in both of the two groups. The incidence of emesis in the probiotics group was also lower than that in the placebo group, without statistically significant difference though. There was no significant difference between the two groups on the incidence of diarrhea also (Supplementary Table 7).

## Discussion

In our study, the probiotic supplement significantly reduced the changes of body weight, BMI, body-fat percentage, plasma LDL, TC, and GLU without any obvious side effects throughout the treatment period. There were 5 metabolites that changed significantly in the two groups, and the change of Biliverdin dihydrochloride was inverse correlated with the change of body weight. For the gut microbiota, after docetaxel-based chemotherapy, *Tenericutes* on phylum level, together with *Bacteroides*, [Eubacterium]_*coprostanoligenes_group*, and *Anaerostipes* on Genus level, showed significant differences in the two groups. Also, the changes of *Bacteroides* and *Anaerostipes* were inverse correlated with the change of LDL. The body weight, BMI, and body-fat percentage of the patients in the probiotics group trended to be lower than those in the placebo group, however, there was no significant difference between the two groups. Our data suggest that probiotics can be potentially used to stabilize the body weight, body-fat percentage, plasma LDL, gut dysbacteriosis, and reduce the metabolic changes during docetaxel-based chemotherapy for breast cancer patients. However, the effectives and long-term effects warranted further studies with a larger sample size.

Docetaxel, one of the taxanes, is a chemotherapeutic drug that targes M phase of cell cycle, and promotes tubulin polymerization while inhibits de microtubule depolymerization, thus it can effectively inhibit cell proliferation and mitosis. Docetaxel is widely used in breast cancer treatment (19). However, the changes of body weight, BMI, body fat, and related factors have been observed to be associated with docetaxel chemotherapy (5, 13, 20, 21). Weight gain during chemotherapy is known as an independent adverse predictor of pCR. Meanwhile, increased body weight and BMI are positively correlated with recurrence rate and mortality of cancer patients (2, 22–25). In our study, the body weight, BMI, and body-fat percentage of patients in the placebo group increased significantly, which is consistent with previous observations.

The mechanism of docetaxel induced changes of body weight, BMI, body-fat percentage, and LDL remains unclear. It may be correlated with the food intake, basal metabolic rate, physical activity, menstrual status, and hormone levels (10–13). It is also known that a nutritional disorder induced by chronic imbalance of energy intake and expenditure is associated with weight gain and obesity (10). Interestingly, in our study, the level of LDL was significantly lower in the probiotics group after docetaxel-based chemotherapy, and the changes of plasma LDL, TC, and GLU in the probiotics group were significantly smaller than the placebo group throughout the treatment period. Furthermore, five plasma metabolites changed differentially in the two groups. In previous studies, plasma methylsuccinic acid was shown to inhibit the normal tricarboxylic acid cycle through acting as the initiating substrate to a variety of methylated tricarboxylic acid cycle metabolites, which are abnormal and potentially harmful (26). Citric acid, an important intermediate in tricarboxylic acid cycle, plays an important role in the metabolism of fatty acids and amino acids (27). It can reflect the function of mitochondria, and has anti-inflammatory and anti-oxidation effects (28). It was found to decrease significantly in the plasma of cancer patients (27). Adenine generally refers to vitamin B4. When it is deficient, it can result in a range of diseases, such as intellectual disability, hematological diseases, dermal diseases, hypoglycemia, immune dysfunction, allergies, and myasthenia (29). It is also associated with lipodystrophy (29). Cholesta-4,6-dien-3-one, one type of oxidized cholesterol ester, and biliverdin dihydrochloride which can reflect the metabolism of cholesterol and bile acid in the body, are associated with a range of diseases (30, 31). In addition, previous study showed that through activating the p38MAPK and p44/42 signaling pathways, probiotic supplement inhibited PPARγ, and alleviated the obesity development and its associated metabolic disorders (32). *Bifidobacterium longum* supplement could increase the level of active ghrelin, leading to an amelioration of deficiencies in ghrelinergic signaling, which is involved in glucose homeostasis and obesity (33). The *Clostridium cochlearium* and *Lactobacillus acidophilus* administration might restored imbalance between the anti-inflammatory and pro-inflammatory states of adipose tissue induced by obesity, leading to an improvement of the insulin sensitivity and glucose homeostasis (34). And *Enterococcus faecalis* can reduce the weight gain induced by the high fat diet by producing propionic acid (PA), a member of short chain fatty acid, which can simulate apoptosis in 3T3-L1 pre-adipocyte (35). These observations are all consistent with our hypothesis that probiotics supplement during docetaxel-based chemotherapy can reduce the change of body weight, BMI, body-fat percentage, plasma LDL, TC, and GLU effectively. Metabolism modulation could be the potential mechanism which needs further studies to investigate.

We also detected the gut microbiota before and after docetaxel-based chemotherapy. Docetaxel administration did not result in any significant changes of gut microbiota on either Alpha or Beta diversity. However, on phylum level, the relative abundance of *Tenericutes* in the probiotics group was significantly higher than the placebo group after docetaxel-based chemotherapy. On Genus level, the relative abundance of *Bacteroides*, [Eubacterium]_*coprostanoligenes_group*, and *Anaerostipes* also showed significant differences in the two groups after docetaxel-based chemotherapy. In previous studies, the abundance of *Tenericutes* was shown higher in diet-induced obese mice (36) or obese Göttingen minipigs (37), which is inconsistent with our study. This may due to the difference of species. Consistent with previous studies, in Göttingen minipigs, obesity induced by certain diet conditions resulted in *Bacteroides* flourishing (37). And [Eubacterium]_*coprostanoligenes_group* is considered to be a cholesterol-reducing bacterium (38). While *Anaerostipes* was shown to be inverse correlated with obesity-related markers in diet-induced obese mice (39). We harvested the stool specimens on the 21st day after the last cycle of docetaxel-based chemotherapy. The gut microbiota dysbiosis associated with docetaxel-based chemotherapy may be partial recovery. However, these results all support that probiotics supplement during docetaxel-based chemotherapy can regulate gut microbiota composition.

Our trial has several limitations. First, as a single-center study with relatively small sample size, the enrolled patients might not fully represent patient population. Second, patients from urban and rural areas have different lifestyle, which might lead to the differences of gut microbiota composition. Third, the effects of diet variation and the different levels of exercise on gut microbiota are still unknown, although we already gave guidance on patients' diet, like they were asked to not take anything contains probiotic or prebiotic ingredients, and their daily diet contains no high fat, high sugar or high salt. They eat three meals a day and each meal contain staple (rice, noodle, steamed bun), meat (pork, beef, lamb, chicken, fish, or seafood) and vegetables. Lastly, the long-term follow-up is not assessed, and the long-lasting protective effects of probiotics is also unknown.

## Data Availability Statement

The original contributions presented in the study are included in the article/Supplementary Material, further inquiries can be directed to the corresponding author.

## Ethics Statement

The studies involving human participants were reviewed and approved by the IRB of Third Xiangya Hospital, Central South University. The patients/participants provided their written informed consent to participate in this study.

## Author Contributions

ZJ, ZQ, BD, and JT: conceptualization and methodology. BD: data curation. BD and JT: formal analysis, funding acquisition, and validation. ZJ, ZQ, and LY: investigation and software. LQ and BD: project administration. LQ, WW, and YW: resources and supervision. ZJ and LY: visualization. ZJ, ZQ, LY, and BD: writing-original draft. All authors: writing-review and editing.

## Funding

This work was supported by Natural Science Foundation of Hunan Province [S2019JJKWLH0198], and National Natural Science Foundation of China [No. 81471107].

## Conflict of Interest

The authors declare that the research was conducted in the absence of any commercial or financial relationships that could be construed as a potential conflict of interest.

## Publisher's Note

All claims expressed in this article are solely those of the authors and do not necessarily represent those of their affiliated organizations, or those of the publisher, the editors and the reviewers. Any product that may be evaluated in this article, or claim that may be made by its manufacturer, is not guaranteed or endorsed by the publisher.

## Abbreviations

HER-2: Human Epidermal Growth Factor Receptor-2
ER: Estrogen receptor
PR: Progesterone receptor
Luminal A: ER (+), and PR≥20%, HER-2 (-), and Ki-67 ≤20%
Luminal B: ER (+), and/or PR (+), HER-2 (-), and Ki-67>20%, or ER (+), and/or PR (+), HER-2 (+)
HER-2-Postive: ER (-), and PR (-), HER-2 (+)
Tri-Negative: ER (-), and PR (-), HER-2 (-)
AJCC: American Joint Committee on Cancer
TNM: Tumor size (T), Nodal status (N), and Metastasis (M)
HIS: Hospital Information System
LC-MS/MS: Liquid Chromatography Coupled with Tandem Mass Spectrometry
rRNA: ribosomal RNA
PCA: Principal Component Analysis
BMI: Body Mass Index
BFP: Body-Fat Percentage
LDL: low density lipoprotein
HDL: high density lipoprotein
TC: total cholesterol
TG: Triglyceride
GLU: blood glucose.
