# Corrigendum: Probiotics for the treatment of docetaxel-related weight gain of breast cancer patients—A single-center, randomized, double-blind, and placebo-controlled trial

**DOI:** 10.3389/fnut.2023.1276556

**Published:** 2023-08-22

**Authors:** Zhang Juan, Zhang Qing, Liang Yongping, Liyuan Qian, Wei Wu, Yanguang Wen, Jianbin Tong, Boni Ding

**Affiliations:** ^1^Department of Breast Surgery, Tangshan People's Hospital, Tangshan, Hebei, China; ^2^Department of Breast and Thyroid Surgery, Third Xiangya Hospital, Central South University, Changsha, China; ^3^Department of Medical Imaging (Ultrasound), Tangshan Central Hospital, Tangshan, China; ^4^Hunan Province Key Laboratory of Brain Homeostasis, Third Xiangya Hospital, Central South University, Changsha, China; ^5^Center for Experimental Medicine, Third Xiangya Hospital, Central South University, Changsha, China; ^6^Department of Anesthesiology, Third Xiangya Hospital, Central South University, Changsha, China

**Keywords:** docetaxel-related weight gain, breast cancer, docetaxel-based chemotherapy, gut microbiota, metabolism

In the published article, there was an error in affiliation [1]. Instead of “Department of Anesthesiology, Third Xiangya Hospital, Central South University, Changsha, China”, it should be “Department of Breast Surgery, Tangshan People's Hospital, Tangshan, Hebei, China”.

The authors apologize for this error and state that this does not change the scientific conclusions of the article in any way. The original article has been updated.

