# Supplementary material for: Probiotics for the Treatment of Docetaxel-Related Weight Gain of Breast Cancer Patients—A Single-Center, Randomized, Double-Blind, and Placebo-Controlled Trial
Source: Front Nutr. 2021 Dec 2;8:762929. doi: 10.3389/fnut.2021.762929 (PMC8675585; doi:10.3389/fnut.2021.762929)
Supplement: Supplementary file 1 [file Data_Sheet_1.DOC]

**Supplementary 1**

**Research Protocol**

**Title:** Probiotics for the Docetaxel-related weight gain of breast cancer patients: A Single-center, Randomized, Double-blind, and Placebo-controlled trial

**Trial site:** Third Xiangya Hospital, Central-South university, Changsha, China.

**Principal Investigator:** Boni Ding

**Version number:** V1·0

**Version date:** May 23, 2018

**STUDY ABSTRACT**

**Design**: A double-blind, randomized controlled trial in Third Xiangya Hospital.

**Aims:** To evaluate the effectiveness of probiotics on protecting Docetaxel-related weight gain of breast cancer patients by conducting a randomized controlled trial.

**Outcome measures:** The primary outcome was the change in body weight and the change in body-fat percentage of the patients enrolled from randomization to the end of the treatment period. Secondary outcomes are as follows: (1) General laboratory test results (routine blood, liver, and kidney function, plasma glucose, fasting insulin, and lipid series); (2) Faecal microbiota compositions before, and after chemotherapy; (3) Plasma metabolite levels before, and after chemotherapy.

**Population:** Newly diagnosed breast cancer (Stage Ⅰ-III) patients (between 20-60 years of age) who had operation needed docetaxel-based chemotherapy (4 cycles of epirubicin at 100 mg/m2 and cyclophosphamidum at 600 mg/m2 followed by 4 cycles of docetaxel at 100 mg/m2 [EC-T]) will be screened for enrollment.

**Eligibility:**

The inclusion criteria are as follows: newly diagnosed breast cancer (Stage I-III) patients who had operation needed docetaxel-based chemotherapy (4 cycles of epirubicin at 100 mg/m2 and cyclophosphamidum at 600 mg/m2 followed by 4 cycles of docetaxel at 100 mg/m2 [EC-T]), between 20-60 years old of age, without immune system diseases, agreed to participate.

The exclusion criteria include: (1) human epidermal growth factor receptor-2 (HER-2) is positive; (2) advanced patients; (3) with other malignant tumors; (4) with diabetes; (5) thyroid dysfunction or after thyroidectomy; (6) pituitary tumor; (7) adrenal gland tumor and other diseases that seriously affected metabolism; (8) history of ovariectomy, thyroidectomy, pituitary surgery, adrenal surgery and other operations that seriously affected endocrine function; (9) history of using antidepressants, weight-loss medications or other medications that promoted weight gain or metabolic loss; (10) participated in or plan to participate in diet or exercise weight loss programs; (11) used antibiotics, probiotics, or gastrointestinal motility drugs within 3 months before admission or during the study; (12) alcoholic or drug addicts; (13) participating in other clinical trials; (14) refused to join the group; (15) could not cooperate with the treatment.

**Treatment:** The intervention includes adjuvant chemotherapy regimen selected by the treating physician. For a total of 84 days, probiotics or placebo will be given 3 capsules BID to probiotics group or placebo group. Patients enrolled will be measured the weight and body fat rate before and after chemotherapy.

**Duration:** The trial will start from Oct, 2018, and sustain at least 12 months.

**1. BACKGROUND & SIGNIFICANCE**

Nowadays, the population of obesity is growing rapidly all over the world . Growing evidence shows that obesity has many effects on human health. Obesity is not only closely related to the metabolic diseases, but also the occurrence of a variety of malignant tumors, together with a worse prognosis .

For women, breast cancer is the most common malignancy , while chemotherapy is one of the main treatments. Docetaxel is one of the most important chemotherapy-agents for chemotherapy of breast cancer. Previous studies showed that it might induce weight gain and the levels of blood glucose, triglyceride, and insulin increasing . Among the breast cancer patients underwent chemotherapy, there were almost 50%-96% emerged varying degrees of weight gain . It is known that weight gain, especially more than 10% of the body weight during the treatment of breast cancer increased recurrent risk and all-cause mortality rates . Those obesity-related diseases (e.g. hypertension, diabetes, cardiovascular and cerebrovascular diseases, gallbladder diseases, etc.) impact on the patients’ quality of life heavily .

The mechanism of Docetaxel-related weight gain remains unclear. It may be related to the change of food intake, basal metabolic rate, physical activity, menstrual status, and hormone levels. It has been suggested that the host gut microbiota is associated with obesity and metabolic syndrome . Probiotics supplement has potential therapeutic effects on weight, metabolic syndrome, and chronic inflammation state .

In this paper, we present a protocol for a randomized controlled trial with probiotics for protecting Docetaxel-related weight gain in Third Xiangya Hospital. Third Xiangya Hospital is a university teaching hospital, and the medical center in the central south part of China, with 2200 beds, more than 100,000 admissions, and more than 73,000 operations per year. We hypothesize that probiotics can prevent Docetaxel-related weight gain.

**2. OBJECTIVES**

To evaluate the effectiveness of probiotics on protecting Docetaxel-related weight gain by conducting a randomized controlled trial in the department of breast, and thyroid surgery in Third Xiangya Hospital.

**3. STUDY DESIGN**

This is a one-arm, parallel group, double-blind, randomized controlled trial in the department of breast, and thyroid surgery in Third Xiangya Hospital.

**4. STUDY APPROACH**

Evaluate the effectiveness of the probiotics’ intervention – A Randomized Trial

**(1) Setting and Participants**

**Setting:** Newly diagnosed breast cancer (Stage Ⅰ-III) patients (between 20-60 years of age) who had operation needed docetaxel-based chemotherapy (4 cycles of epirubicin at 100 mg/m2 and cyclophosphamidum at 600 mg/m2 followed by 4 cycles of docetaxel at 100 mg/m2 [EC-T]) are recruited from 33^rd^ nursing units on the 17^th^ floor in surgical building — department of breast, and thyroid surgery — in Third Xiangya Hospital in Changsha, Hunan, China. Third Xiangya Hospital is an academic medical hospital which has 2200 beds with more than 100,000 admissions, and 73,000 operations per year.

**Inclusion criteria**: Newly diagnosed breast cancer (Stage I-III) patients who had operation, needed docetaxel-based chemotherapy (4 cycles of epirubicin at 100 mg/m2 and cyclophosphamidum at 600 mg/m2 followed by 4 cycles of docetaxel at 100 mg/m2 [EC-T]), between 20-60 years old of age, without immune system diseases, agreed to participate in the study.

**Exclusion criteria**: 1) human epidermal growth factor receptor-2 (HER-2) is positive; 2) advanced patients; 3) with other malignant tumors; 4) with diabetes; 5) thyroid dysfunction or after thyroidectomy; 6) pituitary tumor; 7) adrenal gland tumor and other diseases that seriously affected metabolism; 8) history of ovariectomy, thyroidectomy, pituitary surgery, adrenal surgery and other operations that seriously affected endocrine function; 9) history of using antidepressants, weight-loss medications or other medications that promoted weight gain or metabolic loss; 10) participated in or plan to participate in diet or exercise weight loss programs; 11) used antibiotics, probiotics, or gastrointestinal motility drugs within 3 months before admission or during the study; 12) alcoholic or drug addicts; 13) participating in other clinical trials; 14) refused to join the group; 15) could not cooperate with the treatment.

**(2) Randomization and Concealment**

After admission to the hospital, eligible participants are randomly assigned to probiotics group or placebo group by using SPSS 19.0 software to get random numbers generated in a 1:1 ratio. The randomization is performed by a researcher who is not involved with the intervention or data management or statistical analyses. The results of randomization are sealed in an envelope and stored until the end of the study. Blinding is strictly maintained and is monitored by inspectors (Kaihua Hu). Throughout the trial, all investigators, study participants, staff are blinded to the randomization, and are masked to outcome measurements during the trial. The whole process of the study is monitored by the Institutional Review Board (IRB) of Third Xiangya Hospital.

**(3) Intervention and control**

**Intervention:** Eligible patients are randomly divided into placebo and probiotic groups as described. The probiotic group receive probiotic capsules (0.84 g) twice daily throughout the treatment period. The probiotic capsule (BIFICO, Sine Pharmaceuticals, Shanghai, China) contained Bifidobacterium longum (≥1.0×10^7^ CFU/210mg), Lactobacillus acidophilus (≥1.0×10^7^ CFU/210mg), and Enterococcus faecalis (≥1.0×10^7^ CFU/210mg).

**Control:** The placebo group receive placebo capsules following the same administration schedule. The placebo capsules (provide by Sine Pharmaceuticals, Shanghai, China) contains all ingredients except probiotics, are like the probiotic capsules in shape, size, and smell.

**(4) Implementation procedure**

**Screening patients**

Newly diagnosed breast cancer (Stage Ⅰ-III) patients between 20-60 years old of age, who had operation and needed to undergo docetaxel-based chemotherapy (4 cycles of epirubicin at 100 mg/m2 and cyclophosphamidum at 600 mg/m2 followed by 4 cycles of docetaxel at 100 mg/m2 [EC-T]) will be screened for the eligible patients according to the inclusion criteria. After the introduction of aims and significance of the whole program, patients could decide to accept it or not themselves and sign the consent form if they agree to participate. The registration form for the included patients will be filled in at the same time. According to the randomization grouping methods described above, if the patients randomly enter the intervention group (probiotics group), they will be given probiotics to take until the end of the last cycle of chemotherapy with Docetaxel, and the patients in the control group (placebo group) will be given placebo (look and smell the same with probiotics) to take until the end of the last cycle of chemotherapy with Docetaxel.

**Pre-chemotherapy assessment**

For both groups: Once the chemotherapy schedule is confirmed, the body weight and body-fat percentage of the patients will be measured one day before the first cycle of docetaxel administration twice and take the average and record. The stool and blood specimen will be collected at the same time. Clinical characteristics such as age, height, weight, molecular subtype, disease stage, together with the results of routine blood, liver and kidney function, blood glucose, fasting insulin, and lipid series will be collected from the Health Information System (HIS).

**Under-chemotherapy intervention**

For both groups: Once enrolled in the study, patients will be invited to join a Wechat group. In the Wechat group, besides the patients there are also two professional doctors and one nurse. A volunteer oversees reminding patients to take their medicine. The nurse oversees collecting side effects of chemotherapy. The doctors oversee answering questions from patients enrolled. The results of routine blood, liver, and kidney function, blood glucose, fasting insulin, and lipid series of each cycle of chemotherapy will be collected from the Health Information System (HIS).

**Post-chemotherapy assessment**

For both groups: The body weight and body-fat percentage of the patients will be measured twice, take the average and record again 21 days after the last cycle of docetaxel administration. The stool and blood specimen will be collected at the same time. The results of routine blood, liver and kidney function, blood glucose, fasting insulin, and lipid series of each cycle of chemotherapy will be collected from the Health Information System (HIS).

**The stool and blood specimen collection**

The day before blood collection, patients need to take light food for dinner, and be fasting for more than six hours, avoid strenuous exercise and try to overcome their emotions tension. The fasting blood sample of the patients will be taken by venipuncture and collected using a disposable vacuum tube containing EDTA-K2 anticoagulant one day before the first cycle of docetaxel administration and 21 days after the last cycle of docetaxel administration. The stool samples of patients enrolled will be collected within 20 minutes of production by a researcher using disposable sterile stool specimen box one day before the first cycle of docetaxel administration and 21 days after the last cycle of docetaxel administration. The researcher will transport the stool samples to the laboratory under low temperature within 4 hours after collected, and divided into 2-3 cryopreserved tubes, and froze in the refrigerator (-80℃).

**(5) Assessment and outcome measures**

For the baseline assessment period, all patients undergo pre-chemotherapy assessment one day before the first cycle of docetaxel administration, including an interview, and medical record abstraction by the trained research team. Components of the patient interview consist of: 1) Sociodemographic data; 2) Medical history and comorbidities; 3) The measured data of the body weight and body-fat percentage; 4) Data about surgery and pathological information. The intervention will be lasting until the chemotherapy with Docetaxel ends. Twenty-one days after the last cycle of docetaxel administration another interview will be done. The implementation procedure is shown in Figure 1.


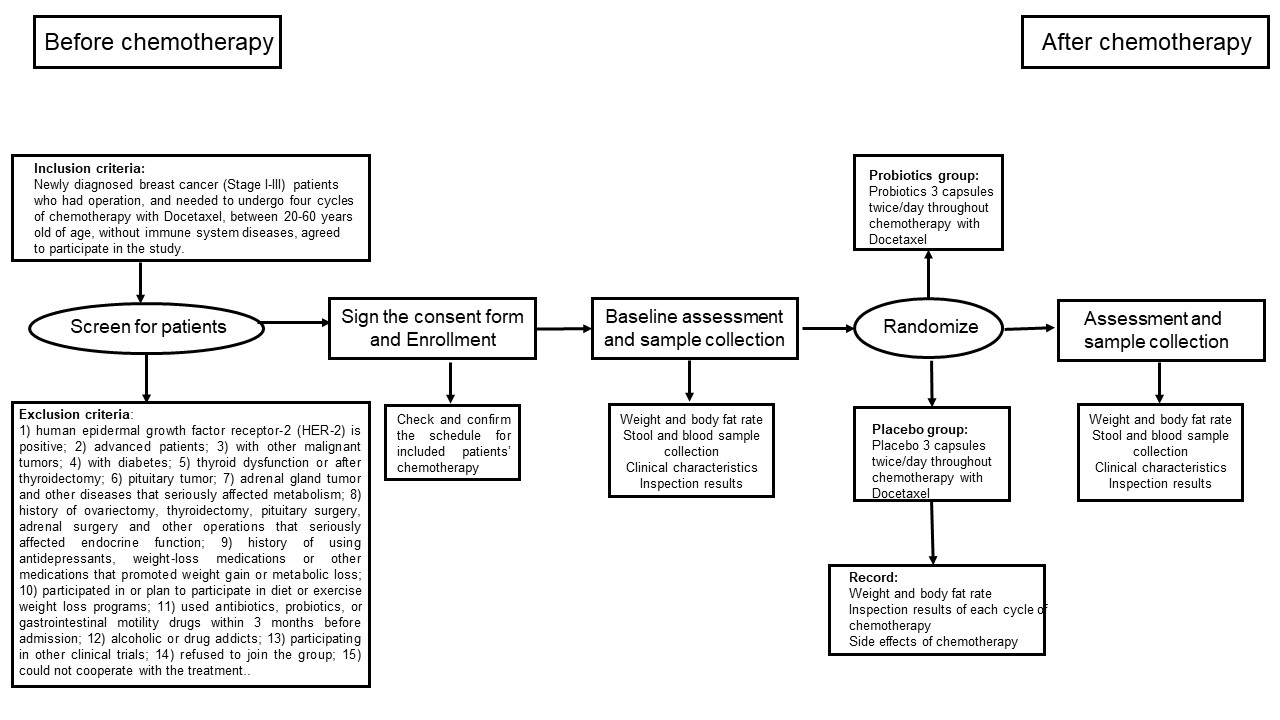
Figure 1. Implementation procedure

**(6) Outcomes**

**Primary Outcome**

The change in body weight and the change in body-fat percentage of the patients enrolled from randomization to the end of the treatment period.

**Secondary Outcome**

a) The effects of docetaxel and/or probiotics on inspection results (including plasma fasting insulin, plasma glucose, and lipid series)

b) The effects of docetaxel and/or probiotics on plasma metabolites;

c) The effects of docetaxel and/or probiotics on gut microbial community.

**Outcome Measures**

The characteristic variables and outcomes will be collected according to the following time schedule using the related tools (Table 1).

| **Table 1 Overview of assessment during the study** | | | | |
| --- | --- | --- | --- | --- |
| **Domain** | **Indicators/Tools** | **Pre** | **During** | **Post** |
| **Demographics** | Age, height, weight | √ | - | √ |
| **Clinical characteristics** | Molecular subtype and disease stage | √ | - | - |
| **Weight and BFP** | Fat analyzer | √ | - | √ |
| **Inspection results** | Routine blood, liver and kidney function, blood glucose, fasting insulin, and lipid series | √ | √ | √ |
| **plasma metabolites** | LC-MS/MS | √ | - | √ |
| **gut microbial community** | Bacterial 16S ribosomal RNA (rRNA) gene sequencing | √ | - | √ |
| Abbreviations: Pre, pre-chemotherapy; During, during chemotherapy; Post, post-chemotherapy; BFP, body-fat percentage; LC-MS/MS, Liquid chromatography coupled with tandem mass spectrometry (LC-MS/MS). | | | | |

**5. STATISTICAL PLAN**

**5.1 Sample size**

In our preliminary experiment, the proportion of patients who gained weight in the placebo group was 85%, and the probiotics group was 40%. According to the equation: $\text{n}=\frac{2\left(\mu_{\alpha}+\mu_{\beta}\right)^{2}p\left(1-p\right)}{\delta^{2}}$, where δ = (P_1_-P_2_), p= (P_1_+P_2_)/2, and P_1_ and P_2_ are the positive rates of the placebo group and the probiotics group, respectively, with significance set at 0.05, and power at 90%, the sample size required to detect a difference is 88 patients. Assuming a loss to follow-up rate of 10%, the total sample size is estimated to be 100 in the study.

**5.2 Statistical analysis**

We plan to perform the analyses by using SPSS, version 19.0 (IBM). To verify the effects docetaxel and/or probiotics on the change in body weight and the change in body-fat percentage of the patients we will use unpaired t test to compare the body weight and body-fat percentage within group and between the two groups. Other variables will be compared using an unpaired t test, or, otherwise, if not normally distributed (such as molecular subtype and disease stage), Mann-Whitney test will be used. All other data with multi-time points will be analyzed with analysis of variance (ANOVA) followed by Scheffe's Test. For the change of plasma metabolites, Chi square test will be used. The Binary Logistic regression and Pearson correlation coefficient will be used to analyze the association of microbiota and metabolites with the primary and secondary outcome. For the gut microbiota, sample alpha-diversity will be summarized using Chao1 estimator and Shannon index, and beta-diversity using Principal Component Analysis (PCA). To compare the gut microbial community, Wilcoxon rank-sum test will be used. A 2-tailed P less than 0.05 is a statistical significance.

**6. DATA MANAGEMENT AND MONITORING**

We will use paper forms for local data collection. The data will be then entered into an Excel form to prepare for further analysis and management. Two research assistants input the data independently to ensure accuracy. We will select 12 cases reported in the database randomly to make sure the consistency by comparing with the original data.

After the data are verified, one special staff member will be keeping the data to make sure no changes are made to the data file. During all preliminary analyses, the analysts, and investigators will be blinded to the results.

**7. ADVERSE EVENTS and MONITORING**

The trial protocol is approved by the Institutional Review Boards (IRB) of Third Xiangya Hospital. All the participants enrolled in this trail will obtain full informed consent before the enrollment.

The Clinical Research Associate (CRA) will monitor adverse events daily. The research team will also monitor the adverse events by daily communication with the patients. If there is any adverse event, the researcher will categorize it as related or not related to the study intervention and report by process. These events are reported in real time to the principal investigators for confirmation and review of grading. All the unanticipated serious adverse events will be reported to the IRB for review regardless of causality. The adverse events will be discussed in the team to make the decision on whether subject participation should be stopped and to make plans for communicating safety concerns to the local healthcare providers. The conclusions and recommendations are summarized in quarterly reports by the project manager. Our study statistician is blinded to subject randomization when analyzing the safety data.

**8. QUALITY CONTROL**

**8.1 Bias**

Before starting the trial, all participants will receive training as required, including theoretical knowledge and how to use the fat analyzer, in order to maintain high consistency of the weight and body fat rate assessment. To minimize error and maximize reliability, the project director will perform the following: (1) providing intensive training to the assessors; (2) monitoring the assessors’ administration of the measurement; (3) meeting with the assessors every week to review procedures and check the quality of the assessment for the primary outcome.

**8.2 Blinding**

We have staff who specialize in randomization, staff who specialize in evaluation, staff who specialize in dispensing, staff who specialize in data entry, and staff who specialize in statistics. During the study ongoing, they are not allowed to communicate for being blinded.

**8.3 Adherence**

As adherence to the intervention is important for assuring the effectiveness of probiotics for protecting Docetaxel-related weight gain in the intervention group, doctors, nurses, and the assessors oversee supervise adherence, and record the actual completion, determine, and record the reasons for non-completion.

**9. APPROVALS AND HUMAN SUBJECT PROTECTION**

This study is approved by the Institutional Review Boards of Third Xiangya Hospital. And applicated for registrant on Dec. 27^th^, 2017, the trial registration number is ChiCTR-INQ-17014181. (http://www.chictr.org.cn/). Before the screening and enrollment, the purpose, process, benefits and risks of the study will be explained to the patients. Make sure that the patients fully understand and will to participate. Written consent is obtained from all trial participants or their next of kin or legal representatives before initiating the trial. All information provided to subjects contains the following elements: title of the study, name of investigator and affiliation, purpose of study, description of procedures, duration of participation, as well as expected risks, inconvenience and benefits.

**Supplementary References**

1 Afshin A, Forouzanfar M H, Reitsma M B, Sur P, Estep K, Lee A *et al.* Health Effects of Overweight and Obesity in 195 Countries over 25 Years. *N Engl J Med*.2017;377: 13-27.doi:10.1056/NEJMoa1614362

2 Demark-Wahnefried W, Peterson B L, Winer E P, Marks L, Aziz N, Marcom P K *et al.* Changes in weight, body composition, and factors influencing energy balance among premenopausal breast cancer patients receiving adjuvant chemotherapy. *J Clin Oncol*.2001;19: 2381-2389.doi:10.1200/jco.2001.19.9.2381

3 Calle E E, Rodriguez C, Walker-Thurmond K,Thun M J. Overweight, obesity, and mortality from cancer in a prospectively studied cohort of U.S. adults. *N Engl J Med*.2003;348: 1625-1638.doi:10.1056/NEJMoa021423

4 Siegel R L, Miller K D,Jemal A. Cancer statistics, 2018.2018;68: 7-30.doi:10.3322/caac.21442

5 Bicakli D H, Varol U, Degirmenci M, Tunali D, Cakar B, Durusoy R *et al.* Adjuvant chemotherapy may contribute to an increased risk for metabolic syndrome in patients with breast cancer. *J Oncol Pharm Pract*.2016;22: 46-53.doi:10.1177/1078155214551315

6 Ingram C,Brown J K. Patterns of weight and body composition change in premenopausal women with early stage breast cancer: has weight gain been overestimated? *Cancer Nurs*.2004;27: 483-490.doi:10.1097/00002820-200411000-00008

7 Playdon M C, Bracken M B, Sanft T B, Ligibel J A, Harrigan M,Irwin M L. Weight Gain After Breast Cancer Diagnosis and All-Cause Mortality: Systematic Review and Meta-Analysis. *J Natl Cancer Inst*.2015;107: djv275.doi:10.1093/jnci/djv275

8 Breast cancer and breastfeeding: collaborative reanalysis of individual data from 47 epidemiological studies in 30 countries, including 50302 women with breast cancer and 96973 women without the disease. *Lancet*.2002;360: 187-195.doi:10.1016/s0140-6736(02)09454-0

9 Demirkan B, Alacacioglu A,Yilmaz U. Relation of body mass index (BMI) to disease free (DFS) and distant disease free survivals (DDFS) among Turkish women with operable breast carcinoma. *Jpn J Clin Oncol*.2007;37: 256-265.doi:10.1093/jjco/hym023

10 Chaudhary L N, Wen S, Xiao J, Swisher A K, Kurian S,Abraham J. Weight change associated with third-generation adjuvant chemotherapy in breast cancer patients. *J Community Support Oncol*.2014;12: 355-360.doi:10.12788/jcso.0078

11 Makari-Judson G, Judson C H,Mertens W C. Longitudinal patterns of weight gain after breast cancer diagnosis: observations beyond the first year. *Breast J*.2007;13: 258-265.doi:10.1111/j.1524-4741.2007.00419.x

12 Saquib N, Flatt S W, Natarajan L, Thomson C A, Bardwell W A, Caan B *et al.* Weight gain and recovery of pre-cancer weight after breast cancer treatments: evidence from the women's healthy eating and living (WHEL) study. *Breast Cancer Res Treat*.2007;105: 177-186.doi:10.1007/s10549-006-9442-2

13 Wang J S, Cai H, Wang C Y, Zhang J,Zhang M X. Body weight changes in breast cancer patients following adjuvant chemotherapy and contributing factors. *Mol Clin Oncol*.2014;2: 105-110.doi:10.3892/mco.2013.209

14 Gérard P. Gut microbiota and obesity. *Cell Mol Life Sci*.2016;73: 147-162.doi:10.1007/s00018-015-2061-5

15 Cerdó T, García-Santos J A, M G B,Campoy C. The Role of Probiotics and Prebiotics in the Prevention and Treatment of Obesity.2019;11.doi:10.3390/nu11030635
