# Supplementary material for: Probiotics for the Treatment of Docetaxel-Related Weight Gain of Breast Cancer Patients—A Single-Center, Randomized, Double-Blind, and Placebo-Controlled Trial
Source: Front Nutr. 2021 Dec 2;8:762929. doi: 10.3389/fnut.2021.762929 (PMC8675585; doi:10.3389/fnut.2021.762929)
Supplement: Supplementary file 3 [file Data_Sheet_3.DOC]

**Article title:**

**Probiotics for the** **Docetaxel-related weight gain of breast cancer patients**

**——A Single-center, Randomized, Double-blind and Placebo-controlled trial**

**Author names:**

Zhang Juan^1^, Zhang Qing^2^, Liang Yongping^3^, Liyuan Qian^2^, Wei Wu^2^, Yanguang Wen^2^, Jianbin Tong^1,4,5^, Boni Ding^2*^

**Affiliation list:**

^1^Department of Anesthesiology, Third Xiangya Hospital, Central South University, Changsha, Hunan, P.R. China

^2^Department of Breast and Thyroid Surgery, Third Xiangya Hospital, Central South University, Changsha, Hunan, P.R. China

^3^Department of Medical Imaging (Ultrasound), Tangshan Central Hospital, Tangshan, Hebei, P.R. China

^4^Hunan Province Key Laboratory of Brain Homeostasis, Third Xiangya Hospital, Central South University, Changsha, Hunan, P.R. China

^5^Center for Experimental Medicine, Third Xiangya Hospital, Central South University, Changsha, Hunan, P.R. China

**^*^Correspondence to:**

Prof. Boni Ding, Department of Breast and Thyroid Surgery, Third Xiangya Hospital, Central South University, 138 Tongzipo Road, Yuelu District, Changsha, 410013, Hunan, P.R. China

Tel: 86-13627418663

Email: [275878133@qq.com](mailto:275878133@qq.com)

**Supplementary Material**

**Table S1. The hematological measurements of participants enrolled over time**

**Table S2.** **The statistics for covariates before the first cycle of docetaxel administration**

**Table S3 ITT analysis of the status of body weight, BMI, and BFP of participants over time**

**Table S4 ITT analysis of the changes of body weight, BMI, and BFP of participants over time**

**Table** **S5**. **ITT analysis of the hematological measurements of participants over time**

**Table S6. The change fold (FC) of metabolites throughout the treatment period**

**Table S7. The incidence of emesis, constipation, and diarrhea in the two groups.**

**Table S1. The hematological measurements of participants enrolled over t**ime

| Results | Probiotics n=47 Mean (SD) | Placebo n=45 Mean (SD) | *P*^b^ value | |
| --- | --- | --- | --- | --- |
| Insulin-pre | 10.80 (6.17) | 11.19 (8.34) | 0.86 |  |
| Insulin-post | 11.36 (8.86) | 11.93 (6.71) | 0.81 |  |
| LDL-pre | 2.55 (1.03) | 2.60 (0.60) | 0.82 |  |
| LDL-post | 2.50 (0.87) | 2.98 (0.68) | 0.005 |  |
| HDL-pre | 1.49 (0.24) | 1.37 (0.20) | 0.08 |  |
| HDL-post | 1.49 (0.29) | 1.42 (0.23) | 0.41 |  |
| TC-pre | 4.89 (1.19) | 4.82 (0.79) | 0.74 |  |
| TC-post | 5.07 (1.08) | 5.35 (0.93) | 0.20 |  |
| TG-pre | 1.54 (1.59) | 1.39 (0.96) | 0.60 |  |
| TG-post | 2.11 (2.15) | 1.70 (0.86) | 0.24 |  |
| GLU-pre | 5.01 (0.91) | 4.96 (0.57) | 0.77 |  |
| GLU-post | 4.95 (0.77) | 5.20 (0.79) | 0.14 |  |
| Δ^a^Insulin | 0.56 (10.11) | 0.75 (8.06) | 0.09 |  |
| Δ^a^ LDL | -0.05 (0.68) | 0.39 (0.58) | 0.002 |  |
| Δ^a^ HDL | -0.001 (0.19) | 0.05 (0.24) | 0.79 |  |
| Δ^a^ TC | 0.18 (0.90) | 0.53 (0.72) | 0.048 |  |
| Δ^a^ TG | 0.57 (1.99) | 0.31 (0.92) | 0.45 |  |
| Δ^a^ GLU | -0.07 (0.71) | 0.23 (0.60) | 0.04 |  |

Abbreviations: LDL, low density lipoprotein; HDL, high density lipoprotein; TC, total cholesterol; TG, Triglyceride; GLU, blood glucose; Pre, one day before the first cycle of docetaxel administration; Post, 21 days after the last cycle of docetaxel administration.

^a^ The changes of hematologic indexes (the level of Post minus Pre)

^b^ The differences between the two groups based on *t* test.

**Table S2.** The statistics for covariates before the first cycle of docetaxel administration

| Variable | Mean Square | *F^a^* | *P^b^* |
| --- | --- | --- | --- |
| Weight | 0.14 | 0.02 | 0.89 |
| BMI | 0.13 | 0.42 | 0.52 |
| BFP, % | 107.77 | 2.03 | 0.16 |
| Insulin | 13.80 | 0.22 | 0.64 |
| LDL | 0.56 | 2.07 | 0.16 |
| HDL | 0.11 | 2.47 | 0.12 |
| TC | 0.52 | 1.44 | 0.24 |
| TG | 1.98 | 2.53 | 0.12 |
| GLU | 0.05 | 0.06 | 0.81 |

Abbreviations: BMI, Body Mass Index; BFP, Body-fat Percentage; LDL, low density lipoprotein; HDL, high density lipoprotein; TC, total cholesterol; TG, Triglyceride; GLU, blood glucose.

^a^ The statistics for covariates before the first cycle of docetaxel administration.

^b^ The *P* value for the effects of the covariates on the results.

**Table S3 ITT analysis of the status of body weight, BMI, and BFP of participants over t**ime

| Results | Probiotics n=50 Mean (SD) | Placebo n=50 Mean (SD) | *P* value |
| --- | --- | --- | --- |
| Weight, mean (SD), kg | 56.18 (6.24) | 58.92 (6.96) | 0.14 |
| BMI, mean (SD) | 22.75 (2.22) | 23.47 (2.71) | 0.30 |
| BFP, mean (SD), % | 31.87 (4.59) | 35.49 (10.20) | 0.10 |
| Weight Change, kg | 0.80 (2.50) | 2.50 (2.95) | 0.03 |
| BMI Change | 0.17 (0.52) | 0.51 (0.60) | 0.04 |
| BFP Change, % | 0.03 (1.10) | 3.50 (10.40) | 0.01 |

Abbreviations: BMI, Body Mass Index; BFP, Body-fat Percentage. The differences of the two groups based on *t* test.

**Table S4 ITT analysis of the changes of body weight, BMI, and BFP of participants over t**ime

| Results | Probiotics n=50 Mean (SD) | | | Placebo n=50 Mean (SD) | | |
| --- | --- | --- | --- | --- | --- | --- |
|  | Pre | Post | *P*^a^ value | Pre | Post | *P*^b^ value |
| Weight, kg | 55.77 (6.48) | 56.18 (6.24) | 0.10 | 57.68 (7.13) | 58.92 (6.96) | <0.001 |
| BMI | 22.57 (2.37) | 22.74 (2.22) | 0.09 | 22.95 (2.78) | 23.47 (2.71) | <0.001 |
| BFP, % | 31.83 (4.65) | 31.87 (4.59) | 0.86 | 32.02 (5.45) | 35.49 (10.20) | 0.03 |

Abbreviations: BMI, Body Mass Index; BFP, Body-fat Percentage; Pre, one day before the first cycle of docetaxel administration; Post, 21 days after the last cycle of docetaxel administration.

^a^ The differences in the probiotics group one day before the first cycle of docetaxel administration and 21 days after the last cycle of docetaxel administration based on *t* test.

^b^ The differences in the placebo group one day before the first cycle of docetaxel administration and 21 days after the last cycle of docetaxel administration based on *t* test.

**Table S5. ITT analysis of the hematological measurements of participants over t**ime

| Results | Probiotics n=50 Mean (SD) | Placebo n=50 Mean (SD) | *P*^b^ value | |
| --- | --- | --- | --- | --- |
| Insulin-pre | 10.49 (6.02) | 15.95 (26.10) | 0.30 |  |
| Insulin-post | 10.84 (8.72) | 11.71 (6.57) | 0.69 |  |
| LDL-pre | 2.26 (0.80) | 2.42 (0.66) | 0.36 |  |
| LDL-post | 2.40 (0.82) | 2.97 (0.67) | 0.03 |  |
| HDL-pre | 1.44 (0.31) | 1.40 (0.21) | 0.61 |  |
| HDL-post | 1.49 (0.28) | 1.46 (0.26) | 0.72 |  |
| TC-pre | 4.75 (0.97) | 5.17 (0.85) | 0.10 |  |
| TC-post | 4.87 (1.00) | 5.37 (0.75) | 0.11 |  |
| TG-pre | 1.32 (0.61) | 1.69 (0.97) | 0.11 |  |
| TG-post | 1.59 (1.23) | 2.10 (1.18) | 0.15 |  |
| GLU-pre | 5.26 (0.62) | 5.14 (1.46) | 0.13 |  |
| GLU-post | 5.06 (0.73) | 5.47 (1.01) | 0.10 |  |
| ΔaInsulin | 0.34 (9.75) | 0.68 (24.99) | 0.38 |  |
| Δa LDL | 0.05 (0.58) | 0.28 (0.52) | 0.01 |  |
| Δa HDL | 0.04 (0.29) | 0.05 (0.23) | 0.88 |  |
| Δa TC | 0.20 (0.64) | 0.57 (0.69) | 0.047 |  |
| Δa TG | 0.28 (0.85) | 0.40 (1.01) | 0.62 |  |
| Δa GLU | -0.20 (0.77) | 0.13 (1.65) | 0.03 |  |

Abbreviations: LDL, low density lipoprotein; HDL, high density lipoprotein; TC, total cholesterol; TG, Triglyceride; GLU, blood glucose; Pre, Pre-chemotherapy; Post, Post-chemotherapy.

^a^ The changes of hematologic indexes (the level of post-chemotherapy minus pre-chemotherapy)

^b^ The *P* value of the differences in the two group.

**Table S6. The change fold (FC) of metabolites throughout the treatment period**

| Results | Probiotics n=47 Mean (SD) | Placebo n=45 Mean (SD) | *P* value |
| --- | --- | --- | --- |
| Methylsuccinic acid | 0.92 (0.12) | 1.24 (0.28) | 0.02 |
| Adenine | 1.96 (0.88) | 0.70 (0.46) | 0.03 |
| Biliverdin dihydrochloride | 0.61 (0.16) | 1.29 (0.92) | 0.047 |
| Citric acid | 1.44 (0.25) | 0.88 (0.20) | 0.02 |
| Cholesta-4,6-dien-3-one | 0.94 (0.20) | 0.57 (0.28) | 0.04 |

**Table S7. The incidence of emesis, constipation,** and diarrhea in the two groups.

| Cycle | Symptom | Probiotics n=47 No. (%) | | | |  | Placebo n=45 No. (%) | | | | | χ^2^ | *P* |
| --- | --- | --- | --- | --- | --- | --- | --- | --- | --- | --- | --- | --- | --- |
|  |  | Grade1 | Grade2 | Grade3 | Grade4 | | | Grade1 | Grade2 | Grade3 | Grade4 |  |  |
| 1 | Emesis | 10 (21) | 8 (17) | 0 (0) | 0 (0) | | | 21 (47) | 11 (24) | 0 (0) | 0 (0) | 1.261 | 0.261 |
|  | Diarrhea | 0 (0) | 0 (0) | 0 (0) | 0 (0) | | | 1 (2) | 0 (0) | 0 (0) | 0 (0) | - | - |
|  | Constipation | 6 (13) | 1 (2) | 0 (0) | 0 (0) | | | 30 (67) | 0 (0) | 0 (0) | 0 (0) | 9.157 | 0.002 |
| 2 | Emesis | 10 (21) | 9 (19) | 0 (0) | 0 (0) | | | 16 (36) | 16 (36) | 0 (0) | 0 (0) | 0.23 | 0.090 |
|  | Diarrhea | 0 (0) | 0 (0) | 0 (0) | 0 (0) | | | 3 (7) | 0 (0) | 0 (0) | 0 (0) | - | - |
|  | Constipation | 6 (13) | 1 (2) | 0 (0) | 0 (0) | | | 30 (67) | 0 (0) | 0 (0) | 0 (0) | 9.157 | 0.002 |
| 3 | Emesis | 11 (23) | 12 (26) | 0 (0) | 0 (0) | | | 15 (33) | 18 (40) | 0 (0) | 0 (0) | 0.035 | 0.851 |
|  | Diarrhea | 0 (0) | 0 (0) | 0 (0) | 0 (0) | | | 1 (2) | 0 (0) | 0 (0) | 0 (0) | - | - |
|  | Constipation | 6 (13) | 1 (2) | 0 (0) | 0 (0) | | | 27 (60) | 3 (7) | 0 (0) | 0 (0) | 0.104 | 0.747 |
| 4 | Emesis | 8 (17) | 14 (30) | 0 (0) | 0 (0) | | | 15 (33) | 18 (40) | 0 (0) | 0 (0) | 0.96 | 0.327 |
|  | Diarrhea | 2 (4) | 0 (0) | 0 (0) | 0 (0) | | | 1 (2) | 0 (0) | 0 (0) | 0 (0) | - | - |
|  | Constipation | 6 (13) | 0 (0) | 0 (0) | 0 (0) | | | 28 (62) | 3 (7) | 0 (0) | 0 (0) | 1.442 | 0.23 |
